# MicroRNAs Regulate Osteogenesis and Chondrogenesis of Mouse Bone Marrow Stromal Cells

**DOI:** 10.4137/grsb.s662

**Published:** 2008-04-22

**Authors:** Salla Suomi, Hanna Taipaleenmäki, Anne Seppänen, Tommi Ripatti, Kalervo Väänänen, Teuvo Hentunen, Anna-Marja Säämänen, Tiina Laitala-Leinonen

**Affiliations:** 1 Bone Biology Research Consortium, Department of Anatomy; 2 Department of Medical Biochemistry and Molecular Biology, Institute of Biomedicine, University of Turku, FI-20520, Finland; 3 Department of Molecular Medicine, National Public Health Institute of Finland; 4 Genome Informatics Unit, Biomedicum Helsinki, FI-00290, Finland

**Keywords:** microRNA, marrow stromal cells, chondrogenesis, osteogenesis

## Abstract

MicroRNAs (miRNAs) are non-coding RNAs that bind to target mRNA leading to translational arrest or mRNA degradation. To study miRNA-mediated regulation of osteogenesis and chondrogenesis, we compared the expression of 35 miRNAs in osteoblasts and chondroblasts derived from mouse marrow stromal cells (MSCs). Differentiation of MSCs resulted in up- or downregulation of several miRNAs, with *miR-199a* expression being over 10-fold higher in chondroblasts than in undifferentiated MSCs. In addition, *miR-124a* was strongly upregulated during chondrogenesis while the expression of *miR-96* was substantially suppressed. A systems biological analysis of the potential miRNA target genes and their interaction networks was combined with promoter analysis. These studies link the differentially expressed miRNAs to collagen synthesis and hypoxia, key pathways related to bone and cartilage physiology. The global regulatory networks described here suggest for the first time how miRNAs and transcription factors are capable of fine-tuning the osteogenic and chondrogenic differentiation of mouse MSCs.

## Introduction

MicroRNAs (miRNAs) are small non-coding RNA-molecules that bind to the 3′ untranslated region of mRNAs and, depending on their degree of complementarity with the target genes, induce translational repression or mRNA degradation.^1^ Since the first identification of miRNAs in 1993,^2^ hundreds of miRNAs have been identified from plants, animals and viruses.^3^ Consecutively, miRNAs have proven to play essential roles in diverse biological processes including early development,^4^,^5^ cell proliferation and cell death,^6^ fat metabolism,^7^ cell differentiation,^8^, ^9^ and brain development.^10^ The sequences coding for miRNAs are spread around the genome, including exons, introns, 3′-UTRs and genomic repeat-areas, and are situated either in the sense or antisense orientation with respect to the overlapping protein-coding gene. Studies carried out with embryonic stem (ES) cells indicate that miRNAs expression profiles in stem cells are different from other tissues,^11^ suggesting that miRNAs may play an important role in stem cell self-renewal and differentiation. The expression of Argonaute genes is restricted to specific anatomical sites in mouse embryos, suggesting that short regulatory RNAs may have physiological functions during organogenesis.^5^ In addition, miRNAs are expressed in haematopoietic tissue where they participate in the regulation of haematopoietic stem cell (HSC) differentiation.^9^, ^12^ However, there is only limited amount of information about the expression of miRNAs in mesenchymal stromal cells (MSCs) or their potential role in osteo- or chondrogenesis.^13^

Mesenchymal stromal cells are multipotent cells that have the potential to differentiate to various lineages of mesenchymal tissues, including bone, cartilage, adipose, tendon, and muscle.^14^ Compared to haematopoietic stem cells (HSCs), MSCs are rare in bone marrow, representing ~1 in 10,000 nucleated cells. Where HSCs are a well-characterized population of self-renewing cells that give rise to all mature blood cell lineages,^15^, ^16^ MSCs are less defined due to the limited understanding of MSC properties. As a result, terms such as “marrow stromal cells”, “mesenchymal progenitor cells”, “nonhaematopoietic mesenchymal stem cells” and “adult nonhaematopoietic stem cells” are used to define this cell population. Isolation and characterisation of stem cells from bone marrow rely on their immunophenotypic or functional aspects. Haematopoietic stem cells have been shown to express surface markers CD14, CD34 and CD45.^17^ MSCs, on the contrary, lack clearly defined surface markers and thus the isolation and characterisation of MSCs is still based on the properties described by Friedenstein already in 1970s: their adherence to plastic, spindle-shaped morphology and ability to form colonies.^18^, ^19^ In addition, MSCs are commonly characterised for their differentiation capacity and for the negative expression of haematopoietic surface markers.

In calcified tissue, MSCs are needed for bone and cartilage formation. During embryogenesis, bone formation begins with mesenchymal stem cell condensation. Membranous bone (craniofacial bones and the clavicle) is derived from MSCs that differentiate *in situ* into bone-forming osteoblasts and produce matrix rich in Type I collagen. Endochondral bone, which is the principal type of bone in the body, is formed by MSCs that first differentiate into chondrocytes to form a cartilagenous template for the bone. Chondrocytes secrete a matrix rich in Type II collagen and Aggrecan, and go through a genetic program driven by Sox9^20^ leading to cartilage enlargement. In the centre of the cartilage anlage, chondrocytes become hypertrophic and start to synthesise Type X collagen that is later degraded and replaced by bone. Although transcription factors such as Sox9 and Runx2, and signalling molecules such as Indian hedgehog (Ihh), Parathyroid hormone-related protein (PTHrP), Fibroblast growth factors (FGF), and Bone morphogenetic proteins (BMPs) are involved in the regulation of endochondral bone formation,^21^ the molecular mechanisms leading to bone formation are still poorly understood. Thus, understanding the regulatory networks that control the lineage commitment and differentiation of MSCs is an important challenge.

In order to study the role of miRNAs in osteo- and chondrogenesis, miRNA expression profiles of osteoblasts and chondroblasts derived from mouse MSCs were compared. Subsequently, target prediction studies carried out with the differentially expressed miRNAs were combined with pathway analyses to gain more insight into the cellular functions potentially regulated by these miRNAs. Bioinformatics studies have shown that the promoter regions of miRNAs seem to contain similar regulatory motifs as the promoter regions of protein coding genes.^22^ In order to investigate whether the studied miRNAs could form regulatory networks with transcription factors (TFs) involved in osteo- or chondrogenesis, the promoter regions of the differentially expressed miRNAs were analysed. We present here multiple lines of evidence to suggest that in addition to haematopoietic cells, miRNAs are also involved in the regulation of lineage commitment in mesenchymal cells.

## Materials and Methods

### Cell culture and RNA extraction

All cell culture reagents, unless otherwise stated, were purchased from Gibco Invitrogen (U.S.A.). Total RNA was extracted from cultured cells before and after osteo- or chondrogenic induction using the mirVana miRNA Isolation Kit following the manufacturer’s protocol (Ambion, U.S.A.). To remove genomic DNA contamination, total RNA samples were digested with DNase I (NEB, U.S.A.). RNA concentrations were quantified using an Eppendorf Biophotometer (Eppendorf, U.S.A.).

Bone marrow cells were isolated from 8–12 week-old male C57BL × DBA mice according to a previously described method.^23^ Briefly, cells were isolated from the tibiae and femora by flushing them from the bone marrow cavity using a 10 ml syringe with a 25 gauge needle and medium consisting of RPMI-1640, 12% iFCS, 100 U/ml penicillin and 100 μg/ml streptomycin. A primary culture of plastic adherent cells from mouse bone marrow is a heterogeneous population of mesenchymal and hematopoietic stem cells.^24^ For the selection of mesenchymal stem cells, bone marrow cells were incubated 2 hours at 37 °C on a plastic culture dish containing RPMI-1640 medium described above (12% iFCS, 100 U/ml penicillin and 100 μg/ml streptomycin) to remove rapidly adherent cells.^18^, ^19^ Unattached cells were collected and cultured in cell culture fl asks at the initial density of 1 × 10^6^ cells/cm^2^. Non-adherent cells were removed 48 hours later and adherent cells were washed with phosphate-buffered saline (PBS). Cells were further cultured with a twice-weekly medium replacement (half of the medium replaced). When confluent, cells were detached using trypsin-EDTA and re-plated at the density of 10 000 cells/cm^2^. RPMI medium has been demonstrated to inhibit the growth of hematopoietic cells in culture^25^ and cultures were therefore maintained in RPMI-1640 for 1 to 2 weeks.^26^ Finally, adherent cells were detached by a trypsin-EDTA treatment and expanded by plating them in DMEM medium supplemented with 12% iFCS, 100 U/ml penicillin and 100 μg/ml streptomycin at the density of 1000 cells/cm^2^. Cells were cultured in described medium until confluent (1 to 2 weeks), thereafter trypsinized, immunophenotypically characterised and subjected to osteoblastic or chondrogenic differentiation.

For immunophenotypic characterisation, MSCs were plated on chamber slides, cultured to confluency and then stained for surface markers Ly-6A/E stem cell antigen 1 (Sca-1) (BD Biosciences, U.S.A.), CD34 (Vision Biosystems, U.S.A.) and CD45 (Dako, Denmark). Cells were incubated for 1 hour with primary antibodies against Sca-1 (1:100), CD45 (1:100) and CD34 (1:400) diluted in DAKO ChemMate Antibody Diluent (Dako, Denmark). Cells were washed with TNT-buffer (0.1 M Tris pH 7.5, 0.15 M NaCl, 0.05% Tween20) and incubated with Post-Blocking reagent (DPVO+500Post, Immunovision Technologies Co., Netherlands) for 20 minutes. Cells were washed three times with TNT buffer and incubated with Poly-HRP anti-Mouse IgG (Immunovision Technologies Co., Netherlands) for 30 minutes. The secondary antibody was detected with DAB (Zymed Invitrogen, U.S.A.) and nuclei were counterstained with Mayer’s hematoxylin.

Osteogenic differentiation was induced by culturing the long-term selected MSCs in osteogenic medium consisting of phenol red-free α-MEM, 12% iFCS, 10 mM Na-β-glycerophosphate (Fluka BioChemika, Switzerland), 50 μgrams/ml ascorbic acid 2-phosphate (Sigma-Aldrich, U.S.A.), 100 U/ml penicillin and 100 μg/ml streptomycin for 3 weeks in cell culture flasks and 24-well plates at the initial density of 10 000 cells/cm^2^. During the first week, the culture medium was supplemented with 10 nM dexamethasone. The cultures were terminated by RNA extraction or fixation in 3% paraformaldehyde. To demonstrate osteoblastic differentiation, cells were stained for alkaline phosphatase (ALP) (Sigma-Aldrich, U.S.A.) and bone nodules were detected by von Kossa staining.^27^ To induce chondrogenic differentiation, 200 000 of the cultured MSCs were placed in a 15-ml polypropylene tube and centrifuged (6 min 500 × g) to form a micro-mass pellet culture.^28^ Cell pellets were cultured for 21 days in chondroinductive medium consisting of high-glucose DMEM supplemented with 10 ng/ml TGF-β3 (R&D Systems, UK), 10^−7^ M dexamethasone, 50 μg/ml ascorbic acid 2-phosphate, 40 μg/ml L-proline (Sigma Aldrich, U.S.A.), 100 μg/ml sodium pyruvate (Sigma Aldrich, U.S.A.), 50 mg/ml ITS+ Premix (BD Biosciences, U.S.A.). Media were changed every 3 to 4 days. After three weeks of culture, the pellets were either lysed for RNA extraction or fixed for 2.5 hours in 4% paraformaldehyde. To evaluate chondrogenic differentiation, pellets were embedded in paraffin, cut into 5 μm sections and stained with toluidine blue for proteoglycans. Presence of type II collagen was detected by 6B3 monoclonal antibody raised against chicken type II collagen^29^ following the method described earlier.^30^

### Osteogenic and chondrogenic gene expression

To further confirm the osteogenic and chondrogenic differentiation of the long-term selected MSCs, the total RNAs were analyzed with RT-PCR for the expression of selected osteogenic and chondrogenic transcripts (Table S1). The osteogenic genes included *Type I collagen*, *Osteocalcin*, *Osterix (Sp7)*, and *Runx2*, whereas chondrogenic differentiation was evaluated based on the expression of *Type II* and *X collagens* and *Sox9*. One μg of DNase I treated total RNA was reverse transcribed using M-MLV reverse transcriptase (Promega Corporation, UK). cDNAs were amplified using DyNAzyme II DNA Polymerase (Finnzymes, Finland) for 35 cycles and analysed on 1,5% agarose gel. Amplification of *GAPDH* and *L19* served as loading controls.

### miRNA expression

A total of 35 miRNAs were selected for the follow-up based on their expression in haematopoietic tissues^9^, ^31^, ^32^ or mouse embryonic stem (ES) cells^11^ or based on computational predictions on physiologically important genes related to bone and cartilage function (Table S2). The expression profiles of these miRNAs were detected by quantitative real-time PCR (qRT-PCR). Amplifications were performed using Taq DNA Polymerase (ABgene) and mirVana qRT-PCR miRNA Detection Kit (Ambion, U.S.A.) following the manufacturer’s instructions. For each sample, a total of 37 different reactions were performed in triplicate with mirVana qRT-PCR Primer Sets. Out of these, 35 were specific for miRNAs and two, U6 snRNA and 5S rRNA, were used for normalisation. For qRT-PCR reactions, 50 ng of DNAse-treated total RNA was used and a no-template reaction was performed for each primer set. RT-reactions, 30 min at 37 °C followed by 10 min at 95 °C, were incubated in MJ Research PTC-200. Real-time PCR reactions were performed in MJ Research PTC-200 DNA Engine Cycler in optical strips. The reactions were incubated at 95 °C for 3 min, followed by 40 cycles of 95 °C for 15 sec and 60 °C for 30 sec. At the end, a dissociation analysis (melt-curve) from 56 °C to 90 °C was performed. Ct data were determined using default threshold settings. End-point reactions were analysed on a 15% neutral PAGE to discriminate between the correct amplification products (~90 bp) and potential primer dimers.

Micro-RNA expression data was normalised to U6 snRNA and 5S rRNA according to the manufacturer’s recommendations. Relative quantification of miRNA expression was calculated with the 2^−ΔΔ^Ct method^33^ where undifferentiated cells were set as a calibrator sample. Standard error of the normalized expression was calculated by applying the differential equation of Gauss.^34^ The miRNA expression values were compared between undifferentiated and differentiated cells, and between osteoblasts and chondroblasts. MicroRNAs, whose expression was changed at least 5-fold or 2-fold, respectively, were selected for target predictions and promoter analyses.

### Target predictions

Target prediction tools TargetScan (http://www.targetscan.org), PicTar (http://pictar.bio.nyu.edu) and miRanda (http://www.microrna.org) were utilized in order to find out possible targets genes for the differentially expressed miRNAs. Since prediction algorithms often result in false positives,^35^ the target gene lists of the three programs were combined and an intersection set, including only the target genes found with all three prediction algorithms, was created for each miRNA separately. Predicted target gene identifiers were first converted into a common nomenclature, and the results were then combined for each miRNA separately using data available from the latest Ensembl release 44.

### Pathway analysis

In order to elucidate the physiological role of differentially expressed miRNAs, their target genes were analyzed through the use of Ingenuity Pathways Analysis version 5.0 (Ingenuity^®^ Systems, www.ingenuity.com). The intersection sets were uploaded into the application, and each gene identifier was mapped to its corresponding gene object in the Ingenuity Pathways Knowledge Base. The genes in the intersection sets were overlaid onto a global molecular network developed from information contained in the Ingenuity Pathways Knowledge Base, and networks were then algorithmically generated based on their connectivity. The functional analysis identified the biological functions or diseases that were most significant to the intersection sets. The significance of the association between the intersection set and pathway was measured in two ways. First, a ratio of the number of genes from the intersection set that mapped to the pathway divided by the total number of genes that mapped to the pathway was calculated. Second, Fischer’s exact test was used to calculate a p-value determining the probability that the association between the genes in the intersection set and the pathway is explained by chance alone. Graphical representations of the molecular relationships between genes/gene products were also produced. Genes/gene products were represented as nodes, and the biological relationship between two nodes was represented as an edge (line). All edges are supported by at least 1 reference from the literature. Nodes were displayed using various shapes that represent the functional class of the gene product.

### Promoter analysis

To examine the potential transcription factors (TFs) involved in miRNA regulation, promoter analysis was performed and conserved binding sites for known TFs were predicted for the upstream regions of differentially expressed miRNA genes. As target prediction algorithms, transcription factor binding site (TFBS) prediction tools often result in false positives. In order to improve the authenticity of the predictions, a technique known as phylogenetic footprinting was applied. Thus, human and mouse orthological sequences were compared and only conserved binding sites were accepted for the analysis. Regulatory regions, 500 bp upstream and 100 bp downstream from the starting locus, of human and mouse orthological pre-miRNAs (Table S3) were retrieved from Ensemble release 44. TFBS predictions were performed by Conreal^36^ using high quality vertebrate matrices from TRANSFAC professional 9.4.^37^, ^38^

## Results

### Cell culture

**Figure 1:**
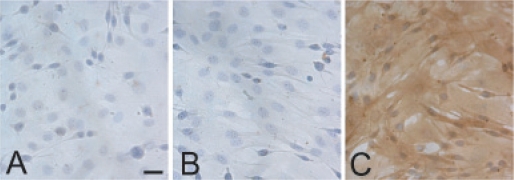
Surface antigen expression in MSCs. Long-term selected MSCs were analysed for the expression of surface antigens CD34 (A), CD45 (B) and Sca-1 (C). Nuclei were counterstained with Mayer’s hematoxylin. Bar = 20 μm.

**Figure 2:**
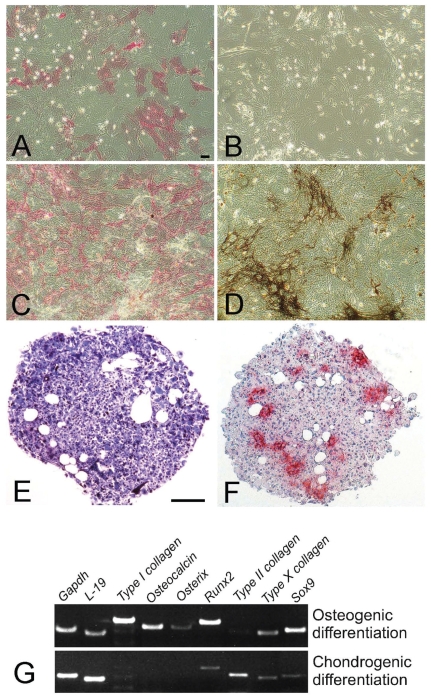
Evaluation of osteogenic and chondrogenic differentiation of MSCs Morphological changes in cells cultured for one (A–B) or three (C–D) weeks in osteogenic medium were evaluated under a light microscope. ALP staining (A and C) was used to visualise osteoblasts and von Kossa staining (B and D) demonstrated calcification in the extracellular matrix. To induce chondroblast differentiation, cell pellets were cultured in chondrogenic medium (E–F). Toluidine blue staining (E) indicated chondrogenic differentiation which was further confirmed by immunohistochemical staining for Type II collagen (red staining in F). Bar = 20 μm in A–D, and 100 μm in E–F. The capacity of long-term selected MSCs for osteogenic and chondrogenic differentiation was evaluated by RT-PCR (G). Total RNA was isolated from cells after osteogenic or chondrogenic differentiation and used for RT-PCR. Amplification products were resolved on a 1,5% agarose gel stained with ethidium bromide. *Gapdh* and *L-19* served as loading controls.

In order to compare miRNA expression in osteoblasts and chondroblasts, mesenchymal cells were isolated and enriched from bone marrow. To confirm the negative selection of HSCs and the positive selection of MSCs, cells were characterised for the expression of CD34, CD45 and Sca-1 surface markers (Fig. 1). After enrichment procedure, cells were positive for stem cell marker Sca-1^39^ and negative for hematopoietic surface antigens CD34 and CD45.^17^ To compare miRNA expression in osteoblasts and chondroblasts, long-term selected MSCs were induced to differentiate into the desired cell types. For osteoblastic differentiation, cells were cultured for one to 3 weeks in osteogenic medium, followed by alkaline phosphatase- and von Kossa stainings (Fig. 2A–D). For chondrogenic differentiation, cells were cultured in chondrogenic medium as micromass pellet cultures for 3 weeks, followed by histological evaluation. Toluidine blue staining demonstrated the presence of proteoglycans and immunohistochemical staining showed deposition of type II collagen in the cell pellets (Fig. 2E–F).

### Osteogenic and chondrogenic gene expression

To further evaluate the phenotypes of the *in vitro* differentiated cell cultures, expression of osteogenic and chondrogenic genes was analysed with RT-PCR (Table S1). After a three-week culture in osteoinductive medium, long-term selected MSCs expressed osteoblast-related genes *Type I collagen*, *Osteocalcin*, *Osterix* and *Runx2*, but also *Type X collagen* and *Sox9*. Cells grown in chondroinductive conditions expressed *Runx2*, *Type II collagen*, *Type X collagen* and *Sox9,* and were negative for the expression of *Type I collagen*, *Osteocalcin* and *Osterix* (Fig. 2G).

### miRNA expression

**Figure 3:**
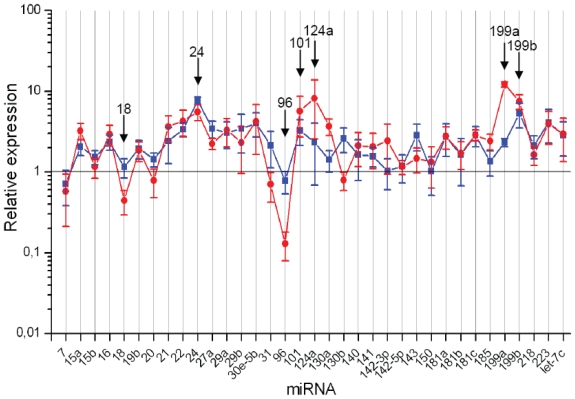
miRNA expression in osteoblasts and chondroblasts derived from MSCs. Relative expression of 35 miRNAs in MSCs after chondrogenic and osteogenic differentiation. 29 miRNAs were selected based on their expression in haematopoietic tissues or ES cells, and 6 miRNAs were selected based on target prediction studies. Expression value 1, which is marked with a black line, represents miRNA expression in undifferentiated precursor cells. miRNA expression after osteogenic differentiation is shown in blue and expression after chondrogenic differentiation is shown in red. The experiment was repeated twice with three replicates for each sample. The data is presented as mean +/− SE. Differentially expressed miRNAs (marked with arrows) were selected for further analysis.

The mirVana qRT-PCR miRNA Detection Kit (Ambion) was used in order to compare miRNA expression in osteoblasts and chondroblasts. Undifferentiated MSCs were used as a control and data were normalized as described in Experimental procedures. Figure 3 shows the miRNA expression profiles of osteoblasts and chondroblasts derived from MSCs. Relative expression value of 1 represents the expression level of a specific miRNA in undifferentiated MSCs. Only 2 out of 35 miRNAs were differentially (≥5-fold) expressed in osteoblasts compared to the undifferentiated cells whereas in chondroblasts the expression levels of 7 out of 35 miRNAs changed at least 5-fold during the differentiation (Table 1). In addition, 8 out of 35 miRNAs were differentially expressed (at least 2-fold) between osteoblasts and chondroblasts derived from long-term selected MSCs (Table 1).

### Pathway analysis

**Figure 4:**
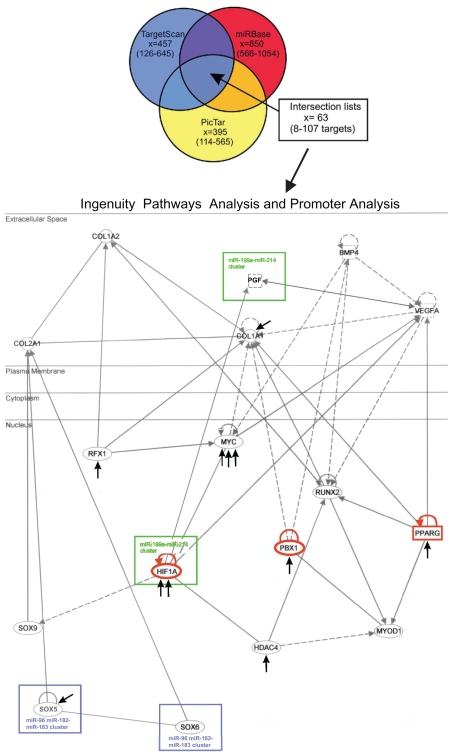
Intersection sets of the differentially expressed miRNAs constitute complex interaction networks. A Venn diagram illustrating the production of intersection sets from three miRNA target prediction algorithms. Differentially expressed miRNAs were selected for miRNA target predictions by PicTar, TargetScan and miRBase. Intersection lists containing 8–107 predicted miRNA target genes (mean 63 genes) were then uploaded to Ingenuity Pathways Analysis (IPA) for further interaction network analysis. The miRNA target genes were overlaid onto a global molecular network developed from information contained in the Ingenuity Pathways Knowledge Base (www.ingenuity.com), and networks were then algorithmically generated based on their connectivity. Results from pathway analysis were combined with promoter analysis data to form a global regulatory network, as shown in the Figure. Composite loops (Shangi et al. 2007) were observed for three TFs marked with red nodes (PBX1, PPARγ and HIF1α); these harboured TFBSs in upstream regions of miRNAs while they were also predicted as target genes for the same miRNA. Two miRNA clusters that were found to target significantly interacting genes are marked in the Figure with rectangles. Arrows represent miRNAs that are predicted to regulate a specific gene. Nodes are displayed using various shapes that represent the functional class of the gene product.

Intersection sets (Fig. 4) of potential miRNA target genes were uploaded into Ingenuity Pathways Analysis version 5.0 (Ingenuity^®^ Systems, www.ingenuity.com), resulting in multiple interaction networks. An exceptionally high score and low p-value, as calculated by IPA, was observed for 5 miRNAs out of 11 and the most significant biological functions related to individual miRNA target gene pathways are shown in Table 2.

### Promoter analysis

Information about eukaryotic transcription factors, their genomic binding sites and DNA-binding profiles is stored in databases such as TRANS-FAC^37^ and JASPAR.^40^ The upstream regions of differently expressed miRNA genes (Table S2) were analysed in order to study the potential transcription factors (TFs) involved in miRNA regulation. When the results were combined with data obtained from target predictions and IPA analyses, it could be noted that 3 transcription factors (PBX1, PPARγ and HIF1α) that were predicted as targets for the differentially expressed miRNAs had also binding sites in the upstream region of the same miRNAs (Table S4). PBX1 (pre-B-cell leukemia homeobox) is a potential target for *miR-101* that was over 5-fold upregulated in chondroblasts. PPARγ (peroxisome proliferator-activated receptor γ) is potentially regulated by *miR-130b* that came up when comparing the miRNA expression between osteoblasts and chondroblasts. HIF1α was predicted to be regulated by *miR-199a* that was over 12-fold upregulated in chondroblasts.

When the interplay of miRNA target genes and TFs was analysed on the basis of published observations, a global regulatory network could be observed (Fig. 4B). *miR-199a* that was upregulated in chondroblasts was found to target HIF1α *miR-124a* was also upregulated in chondroblasts with RFX1 as its target. *miR-96* expression was strongly suppressed in chondroblasts, and it was found to target *SOX5*, a transcription factor that controls chondrogenesis. The previously described cartilage-specific *miR-140* target HDAC4^41^ could also be explained by the regulatory network presented in Figure 4, yet the expression of *miR-140* remained constant in our experiments. When the existence of genomic miRNA clusters were analysed for the miRNAs studied here, two miRNAs were found to be located in clusters, namely ***miR-199a****/miR-214* and ***miR-96****/miR-182/miR-183*. These clusters may strengthen the regulation of Sox5-Sox6 axis leading to type II collagen responses, or of HIF1α-PGF-axis leading to various hypoxia responses (Fig. 4).

## Discussion

Although miRNAs have been shown to play an important role in cell differentiation, their contribution to osteo- or chondrogenesis has not been previously demonstrated. We present here multiple lines of evidence to suggest that miRNAs are an integral part of the transcription factor network regulating bone marrow stem cell differentiation and proliferation. Promoter analyses and target predictions carried out with differentially expressed miRNAs show that transcription factors may act as direct miRNA targets, but also as upstream regulators of miRNA target genes, or the miRNAs themselves. This way they constitute loops that strengthen or attenuate regulatory events.

Since there was no previous data about miRNA expression in mesenchymal stromal cells, we selected the miRNAs for this experiment based on their expression in haematopoietic tissue. We hypothesised that the haematopoietic miRNAs may also target genes involved in the differentiation of mesenchymal tissue within the bone marrow microenvironment. Haematopoietic miRNAs were supplemented with miRNAs found from mouse embryonic stem cells and with miRNAs selected based on target prediction studies carried out with genes involved in bone or cartilage function. In addition, *miR-140* was included based on its suggested role in chondrogenesis.^41^

To gain more insight into the cellular functions possibly affected by the studied miRNAs, the predicted target genes of the differently expressed miRNAs were analysed. We hypothesised that if miRNA target genes are physiologically relevant, they should produce significant interaction networks in the pathway analysis. Extremely low p-values and extensive interactions between genes predicted as miRNA targets were observed, which cannot be explained by chance alone. Bioinformatics’ approaches have suggested that miRNA expression may be regulated by transcription factors.^42^ When the interaction networks between miRNAs and transcription factors were computationally studied, thousands of human genes were suggested to be regulated by miRNA-TF interactions.^43^ As miRNAs are known to target many TFs, the regulatory network appears to be very complex. Composite loops^43^ were observed in our analysis for 3 TFs: PBX1, PPARγ and HIF1α. These harboured binding sites in the upstream region of miRNAs while they were also predicted as target genes for the same miRNA. In addition, numerous TF binding sites were observed in the upstream regions of the differentially expressed miRNAs.

**Figure 5:**
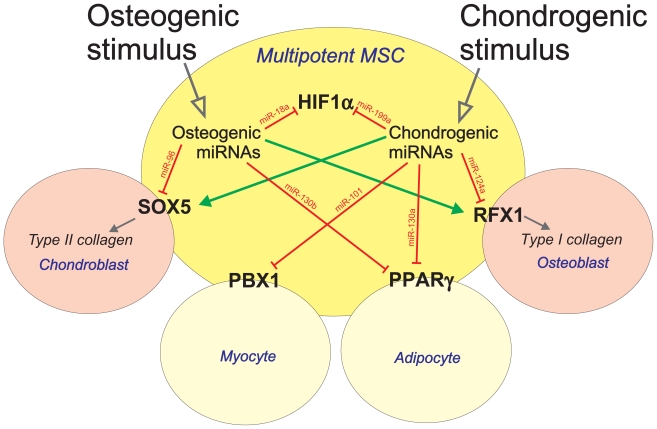
Model for miRNA-TF interactions during the differentiation of bone marrow stromal cells into bone-forming osteoblasts or chondroblasts. Multiple lines of evidence suggest that miRNAs substantially downregulated in chondroblasts targeted genes important for chondrogenic differentiation. In addition, miRNAs significantly upregulated in chondroblasts appeared to target genes important for osteogenesis, adipogenesis and myogenesis. PPARγ (involved in adipogenesis) was targeted by *miR-130a* and *miR-130b* that were upregulated in chondroblasts and osteoblasts, respectively. PBX1 (involved in myogenesis) is a potential target for *miR-101* that was over 5-fold upregulated in chondroblasts. Osteoblasts were positive for *miR-96* that downregulated Type II collagen expression via Sox5. Chondroblasts were positive for *miR-124a* that regulates Type I collagen expression via RFX1. HIF1α was targeted both by *miR-18a* (expressed in osteoblasts) and *miR-199a* (expressed in chondroblasts), suggesting that hypoxic signals are important regulators of MSC differentiation. At the tissue level significant physiological responses may be seen already by affecting the balance between these miRNAs and their target genes. How these regulatory pathways that seem to be important in MSC differentiation can be utilised e.g. in regenerative medicine remain to be resolved.

It became obvious from these analyses that only a fraction, if any, of the miRNA responses are such that lead to a single, easily predictable outcome in cells. Instead, accumulation of several miRNA responses eventually may lead to physiological responses (Fig. 5). An example of such a response is the downregulation of Type I collagen expression by *miR-124a*. The TF RFX1 (Regulatory factor X1) is a target of *miR-124a*, which was upregulated in chondroblasts. Another example is Sox5, which regulates Type II collagen expression during chondrogenesis but also lipase expression, raising the possibility that Sox5 has also a role in adipogenesis and lineage commitment. In MSC cultures, β-glycerophosphate and ascorbic acid induce osteoblast formation and lead to *miR-96* upregulation. During osteoblastic differentiation, adipocyte or chondrocyte genes, like Sox5-regulated lipase or Type II collagen, must be turned off e.g. by *miR-96* in favour of osteogenic differentiation. The complex regulatory networks described here indicate that miRNA-TF interactions are powerful modulators of bone marrow stromal cell differentiation. The specific biological functions related to the target genes of differentially expressed miRNAs suggest that they are involved in developmental pathways including cellular development and movement, cell morphology, cell signalling, cell death, and connective tissue development and function.

## Supplementary Material

**Table S1:** PCR primers used for osteogenic and chondrogenic gene amplification.

Gene symbol	Forward primer	Reverse primer	Amplicon size
*GAPDH*	aggtgaaggtcggagtcaacg	gctcctggaagatggtgatgg	232 bp
*RPL19*	ctgaaggtcaaagggaatgtg	ggacagagtcttgatgatctc	195 bp
*COL1A1*	gaagtcagctgcatacac	aggaagtccaggctgtcc	312 bp
*BGLAP (Osteocalcin)*	ctgctcactctgctggccctgg	ggcggtcttcaagccatactgg	243 bp
*SP7 (Osterix)*	actcatccctatggctcgtg	ggtagggagctgggttaagg	238 bp
*RUNX2*	ccgcacgacaaccgcaccat	cgctccggcccacaatctc	289 bp
*COL2A1*	agagacctgaactgggcaga	gcaccattgtgtaggacacg	201 bp
*COL10A1*	cctgcagcaaaggaaaactc	tggcttaggagtgggagcta	179 bp
*SOX9*	cgactacgctgaccatcaga	agactggttgttcccagtgc	188 bp

**Table S2:** MicroRNA predictions on target genes related to osteoblast and chondrocyte function.

Target gene	Description	miRNA ID
***Osteoblastic genes***		
*COL1A1*	Type I collagen	*miR-29a, miR-150, miR-185*
*BGLAP*	Osteocalcin	*–*
*RUNX2*	Runt-related transcription factor	*–*
***Chondrogenic genes***		
*COL2A2*	Type II collagen	*miR-7, miR-29a, miR-29b*
*COL10A1*	Type X collagen	*miR-101*
*SOX9*	SRY (sex determining region Y)-box 9	*miR-101, miR-124*

Target predictions for the selected genes were carried out with TargetScan, PicTar, or miRanda miRNA target prediction tools.

**Table S3:** Human and mouse pre-miRNAs that were used for promoter analysis described in Experimental procedures.

miRNA ID	Accession *(H. sapiens)*	Chromosome coordinates *(H. sapiens)*	Overlapping transcripts *(H. sapiens)*	Orthologous sequence locus *(M. musculus)*
*hsa-mir-18*	MI0000072	13: 90801006–90801076 [+]	Sense; 2	14: 113925688–113925783 [+]
*hsa-mir-24–1*	MI0000080	9: 96888124–96888191 [+]	Sense; many	13: 63310430–63310497 [+]
*hsa-mir-24–2*	MI0000081	19: 13808101–13808173 [−]	Sense; 1	8: 87098920–87099026 [+]
*hsa-mir-31*	MI0000089	9: 21502114–21502184 [−]	Sense; 1	4: 88381788–88381893 [−]
*hsa-mir-96*	MI0000098	7: 129201768–129201845 [−]	Intergenic	6: 30119456–30119561 [−]
*hsa-mir-101–1*	MI0000103	1: 65296705–65296779 [−]	Intergenic	4: 100844877–100844959 [−]
*hsa-mir-101–2*	MI0000739	9: 4840297–4840375 [+]	Sense; many	19: 29201276–29201372 [+]
*hsa-mir-124a–1*	MI0000443	8: 9798308–9798392 [−]	Intergenic	14: 63544767–63544851 [+]
*hsa-mir-124a–2*	MI0000444	8: 65454260–65454368 [+]	Intergenic	3: 17987813–17987921 [+]
*hsa-mir-124a–3*	MI0000445	20: 61280297–61280383 [+]	Antsense; 1	2: 180823448–180823515 [+]
*hsa-mir-130a*	MI0000448	11: 57165247–57165335 [+]	Intergenic	2: 84541954–84542017 [−]
*hsa-mir-130b*	MI0000748	22: 20337593–20337674 [+]	Intergenic	16: 17037624–17037705 [−]
*hsa-mir-142–3p*	MI0000458	17: 53763592–53763678 [−]	Intergenic	11: 87573059–87573122 [+]
*hsa-mir-199a–1*	MI0000242	19: 10789102–10789172 [−]	Antisense; many	9: 21246897–21246966 [−]
*hsa-mir-199a–2*	MI0000281	1: 170380298–170380407 [−]	Antisense; many	1: 164054491–164054600 [+]
*hsa-mir-199b*	MI0000282	9: 130046821–130046930 [−]	Antisense; many	2: 32140469–32140578 [+]

**Table S4:** Biological roles for TFs with binding sites upstream of miRNA target genes. The highlighted TFs represent composite loops where the TF binds to a miRNA but is also a target for the same miRNA. All interactions are supported by published data, with key references listed below.

miRNA(s)	TF	Downstream target gene(s)	Physiological response(s)	Reference(s)
*miR-101*	**PBX1**	*MyoD, COL1A1, BMP4*	Suppression of myogenic differentiation in favour of osteogenesis or chondrogenesis. PBX1 is regulated e.g. by retinoic acid.	Maves et al. (2007) Development 134:3371–3382
*miR-130b*	**PPARγ**	Multiple adipocyte genes, *PPAR*→*UCP1*→ *NRF1*	Suppression of adipogenic differentiation in favour of osteogenesis or chondrogenesis. PPARγ is regulated e.g. by dexamethasone and NFκB.	Hong et al. (2005) Science 309:1074–1078
*miR-199a*	**HIF1α**	*SOX9, HDAC4, RUNX2, ARNT*	Regulation of cell differentiation and proliferation in hypoxia. The major regulator of HIF1α is oxygen.	Wang et al. (2007) J Clin Invest 117:1616–1626; Schipani et al. (2001) Genes Dev15:2865–2876
*miR-124a*	**RFX1**	*Type I collagen*	Suppression of osteogenic differentiation in favour of chondrogenesis. RFX1 is regulated e.g. by interferon gamma.	Xu et al. (2006) J Biol Chem 281:9260–9270
*miR-96*	**SOX5**	*Type II collagen*	Supression of chondrogenic differentiation in favour of osteogenesis. SOX5 is regulated e.g. by NFκB, retinoic acid and CDC42.	Lefebvre et al. (1998) EMBO J 17:5718–5733; Smits et al. (2001) Dev Cell 1:277–290

Abbreviations: PBX, pre-B-cell leukemia homeobox 1; PPARγ, peroxisome proliferator-activated receptor γ; HIF1α, hypoxia-inducible factor 1α; RFX1, Regulatory factor X1; SOX5, sex determining region Y-box 5.

## Figures and Tables

**Table 1:** Differentially expressed miRNAs in MSC-derived osteoblasts (O) and chondroblasts (C).

*Comparison of osteoblasts and chondroblasts to MSCs (at least 5-fold difference)*			
Osteoblasts	Expression relative to MSCs	Chondroblasts	Expression relative to MSCs
*miR-24*	7,66	*miR-18*	0,44 ↓
*miR-199b*	5,30	*miR-24*	5,54
		*miR-96*	0,13↓
		*miR-101*	5,66
		*miR-124a*	8,18
		*miR-199a*	12,13
		*miR-199b*	7,46
***Comparison between osteoblasts and chondroblasts (at least 2-fold difference)***			
**O vs. C**	**Ratio**	**C vs. O**	**Ratio**

*miR-18*	2,65	*miR-124a*	3,50
*miR-31*	3,05	*miR-130a*	2,61
*miR-96*	5,73	*miR-142-3p*	2,35
*miR-130b*	3,31	*miR-199a*	5,25

**Table 2:** Systemic analysis of specific biological functions related to predicted miRNA target genes.

miRNA	Specific biological functions	−log(p-value)	Focus genes
*miR-96*	Developmental disorder	5,0	13
	Cellular movement	4,1	10
	Cellular assembly and organisation	4,0	18
	Cell morphology	4,0	17
	Connective tissue development and function	4,0	10
	Cancer	3,2	14
	Small molecule biochemistry	3,0	19
	Lipid metabolism	3,0	11
	Genetic disorder	2,5	10
*miR-124a*	Cancer	3,5	11
	Cellular development	3,0	16
	Cellular movement	2,5	14
*miR-130a*	Cellular movement	5,2	13
	Cell signalling	3,3	18
	Gene expression	3,0	14
*miR-130b*	Cell cycle	4,5	12
	Cell death	3,5	15
	Cellular movement	2,8	12
*miR-199a*	Cell death	4,9	10
	Cancer	3,0	10

A total of 79 biological functions were evaluated for each miRNA intersection set by Ingenuity Pathways Analysis. The statistically significant biological functions with 10 or more focus genes (target genes of a given miRNA) are listed here.
